# Bioconversion of Milk Permeate with Selected Lactic Acid Bacteria Strains and Apple By-Products into Beverages with Antimicrobial Properties and Enriched with Galactooligosaccharides

**DOI:** 10.3390/microorganisms8081182

**Published:** 2020-08-03

**Authors:** Egle Zokaityte, Darius Cernauskas, Dovile Klupsaite, Vita Lele, Vytaute Starkute, Paulina Zavistanaviciute, Modestas Ruzauskas, Romas Gruzauskas, Grazina Juodeikiene, João Miguel Rocha, Saulius Bliznikas, Pranas Viskelis, Romas Ruibys, Elena Bartkiene

**Affiliations:** 1 Institute of Animal Rearing Technologies, Faculty of Animal Sciences, Lithuanian University of Health Sciences, Mickeviciaus str. 9, LT-44307 Kaunas, Lithuania; egle.zokaityte@lsmuni.lt (E.Z.); darius.cernauskas@lsmuni.lt (D.C.); dovile.klupsaite@lsmuni.lt (D.K.); vita.lele@lsmuni.lt (V.L.); vytaute.starkute@lsmuni.lt (V.S.); paulina.zavistanaviciute@lsmuni.lt (P.Z.); saulius.bliznikas@lsmuni.lt (S.B.); 2 Department of Food Safety and Quality, Faculty of Veterinary, Lithuanian University of Health Sciences, Mickeviciaus str. 9, LT-44307 Kaunas, Lithuania; 3 Food Institute, Kaunas University of Technology, Radvilenu rd. 19, LT-50254 Kaunas, Lithuania; 4 Department of Anatomy and Physiology, Faculty of Veterinary, Lithuanian University of Health Sciences, Mickeviciaus str. 9, LT-44307 Kaunas, Lithuania; modestas.ruzauskas@lsmuni.lt; 5 Institute of Microbiology and Virology, Faculty of Veterinary, Lithuanian University of Health Sciences, Mickeviciaus str. 9, LT-44307 Kaunas, Lithuania; 6 Department of Food Science and Technology, Kaunas University of Technology, Radvilenu rd. 19, LT-50254 Kaunas, Lithuania; romas.gruzauskas@ktu.lt (R.G.); grazina.juodeikiene@ktu.lt (G.J.); 7 REQUIMTE–Rede de Química e Tecnologia, Laboratório de Química Verde (LAQV), Departamento de Química e Bioquímica, Faculdade de Ciências da Universidade do Porto (FCUP), Rua do Campo Alegre, s/n., P-4169-007 Porto, Portugal; 8 Institute of Horticulture, Lithuanian Research Centre for Agriculture and Forestry, Kauno str. 30, LT-54333 Babtai, Lithuania; biochem@lsdi.lt; 9 Institute of Agricultural and Food Sciences, Agriculture Academy, Vytautas Magnus University, K. Donelaicio str. 58, LT-44244 Kaunas, Lithuania; romas.ruibys@vdu.lt

**Keywords:** milk permeate, lactic acid bacteria, galactooligosaccharides, antimicrobial properties, fermented beverages, apple by-products

## Abstract

The present research study aims to prepare prototypes of beverages from milk permeate (MP) using fermentation with 10 different strains of lactic acid bacteria (LAB) showing antimicrobial properties (*L*. *uvarum* LUHS245, *L. casei* LUHS210, *L. curvatus* LUHS51, *L. plantarum* LUHS135, *P. acidilactici* LUHS29, *L. plantarum* LUHS122, *L. coryniformins* LUHS71, *L. paracasei* LUHS244, *P. pentosaceus* LUHS183, *L. faraginis* LUHS206) and MP with (AppMP) or without (MP) the addition of 8% (*w/w*) apple by-products (App). Two groups of prototypes of beverages were prepared: fermented MP and fermented MP with App (AppMP). Acidity parameters, LAB viable counts, lactose and galactooligosaccharides (GOSs) content, antimicrobial properties against 15 pathogenic and opportunistic bacterial strains, overall acceptability and emotions induced of the final fermented beverages for consumers were evaluated. Results showed that all LAB grew well in MP and LAB strain exhibited a significant (*p* ≤ 0.05) influence on galactobiose and galactotriose synthesis in the fermentable MP substrate. The highest total content of GOS (26.80 mg/100 mL) was found in MPLUHS29 fermented beverage. In addition, MPLUHS245, MPLUHS210 and AppMPLUHS71 fermented beverages showed high antimicrobial activity, inhibiting 13 out of 15 tested microbial pathogens. The overall acceptability of AppMP fermented beverages was 26.8% higher when compared with fermented beverages without App (MP), and the most intensive “happy” emotion was induced by MPLUHS71, MPLUHS24, MPLUHS183 and MPLUHS206 samples. Finally, very promising results were also attained by the bioconversion of MP with selected LAB and App addition into the prototypes of antimicrobial beverages enriched with GOS.

## 1. Introduction

World’s industry is moving towards “zero waste economy”, which promotes the use of by-products as raw materials for the creation of new functional products with high added value. The objective of this industrial switch, based on the circular economy approach, is to reduce losses of valuable functional and bioactive materials and, simultaneously, to overcome management, economic and environmental handicaps during technological implementation through the utilization and valorization of agro-food industry by-products [1]. Most of the technological prototypes for the by-product valorization include expensive biorafination or extraction schemes. These systems present often, at industrial scale, technical, economic feasibility and environment sustainability bottlenecks to be overcome. Unfortunately, some available extraction and recovery procedures are frequently more impacting to the environment than the conventional solutions. In addition, these technological solutions may present scale-up limitations and/or are difficult to be integrated into conventional industrial systems.

One of the challenges of dairy industry is to find an answer to the large quantities of dairy by-products, e.g., the milk permeates received considerable attention as suitable carbon sources, since they are substrates which do not require extensive purification, and may even be the most appropriate option for the development of novel products with added value. Many of cheese whey and second cheese whey products (e.g., lactic acid, bioalcohols, and biogases) have been extensively studied and reviewed over time as possible solution for cheese whey valorization because of their high industrial interest [2]. However, studies about milk permeate valorization are scarce. Milk permeate (i.e., the semi-skimmed milk or the skimmed milk) is obtained after elimination by ultrafiltration of milk proteins and dairy fat contained in the milk. Membrane filtration processes generate high volumes of milk permeate that contains lactose, minerals and serum proteins in their native’s state, if the previous milk was not subject to heat treatments. Compared with cheese whey, which is the main by-product of dairy industry used for the production of value-added compounds [3], the milk permeates are free of the rennet by-product glycomacropeptides, residual fats and microorganisms (phage, cellular debris, etc). Due to their composition and the native state of their proteins, milk permeates are useful fluids to prepare beverages with desirable nutritional properties and high functional value, as well as in meeting the demands of contemporary consumers for natural food products that can effectively promote a healthy life-style and well-being. Despite these advantages, permeate pH is similar to that of milk and due to the poor sensory evaluation, it is not acceptable for most commercial applications. Although unprocessed permeate is not an appealing and useful food product by itself, it is possible however to improve its physicochemical, antimicrobial (resistance against food spoilage microorganisms) and sensory quality, and food safety through its fermentation with selected lactic acid bacteria (LAB) strains and adding natural flavoring additives, e.g., apple juice preparation by-products—which demonstrated very strong antimicrobial and antifungal properties in previous studies [4].

LAB strains are used as microbial starter cultures for food fermentation because they display important beneficial effects with high interest in food industry for several reasons. Primarily, LAB can enhance flavor, texture and nutritional value of food products [5,6]. In addition, LAB take a leading role in food fermentation as they not only improve sensory properties of foods, but also increase its biosafety [7]. Most of the LAB strains possess antimicrobial properties, because of various metabolic processes—for example lactose metabolism, bacteriocin and bacteriocin-like inhibitory substance (BLIS) production, organic acids (mainly lactic and acetic acids), polysaccharide biosynthesis, proteolytic enzymes, bacteriophage resistance, metal-ion and antibiotic resistance, and citrate uptake [8,9,10,11,12,13,14,15,16,17]. Incorporation of fermented foods is very important towards a healthy and balanced diet, since fermented foods offer numerous benefits to human health [18].

One of the main functions of LAB in industrial dairy fermentation is lactose utilization [3,19]. Besides, lactose intolerance affects about 70% of the world population and lactose fermentation is an important and beneficial process to reduce or eliminate lactose content in food. Indeed, lactose intolerance results in many gastrointestinal problems, such as bloating, flatulence, diarrhea and abdominal discomfort [3,20]. In fermentation processes, LAB may convert lactose into functional compounds such as galactooligosaccharides (GOSs). Edible galactooligosaccharides are composed by chains of 2 to 9 galactose units and a terminal glucose. GOSs are considered prebiotic compounds [3,21], since they pass undigested through the small intestine in humans to reach the colon. There these prebiotics become selectively metabolized by some beneficial intestinal bacteria population, such as bifidobacteria and lactobacilli (probiotics), thus promoting their growth and multiplication and their bioactivity results in the production of beneficial microbial metabolites (postbiotics) not only to humans but also to animals and fishes. GOSs may protect against intestinal pathogens via their antiadhesive activity of enteric pathogens on the surface of gastrointestinal epithelial cells [11,22,23].

The nutritional value of fermented milk permeates can be further improved by adding natural functional additives. An example of functional additives are the preparations of apple juice by-products. Our previous studies revealed that apple juice by-product preparations are suitable ingredients to design antimicrobial food and feed products with a wide spectrum of inhibition capacity against pathogenic and opportunistic bacterial strains [6,11,24]. Finally, we hypothesized that the conception of a straight bioconversion of milk permeates with selected LAB strains to produce beverages enriched with GOS and possessing antimicrobial capacities is possible in an environmentally sustainable and cost-effective manner. Besides, apple juice preparation by-products can be also included in beverages formulas as flavoring and antimicrobial/antifungal ingredients.

The aim of the current study was to prepare prototypes of milk permeate-based (MP) functional beverages and resorting to LAB strains with antimicrobial properties (*Lactobacillus uvarum* LUHS245, *L. Lactobacillus casei* LUHS210, *Lactobacillus curvatus* LUHS51, *Lactobacillus plantarum* LUHS135, *Pediococcus acidilactici* LUHS29, *Lactobacillus plantarum* LUHS122, *Lactobacillus coryniformins* LUHS71, *Lactobacillus paracasei* LUHS244, *Pediococcus pentosaceus* LUHS183 and *Lactobacillus faraginis* LUHS206) and with (AppMP) or without (App) apple juice by-product preparations. During the research experiments, acidity parameters, LAB viable counts, lactose concentration, content galactooligosaccharide with different degrees of polymerization (2 to 4), antimicrobial properties, and overall acceptability and emotions induced for consumers by testing the novel developed beverages were evaluated.

## 2. Materials and Methods

### 2.1. Lactic Acid Bacteria Strains, Milk Permeate and Apple by-Products Used in the Preparation of Beverages

The *L. uvarum* LUHS245, *L. casei* LUHS210, *L. curvatus* LUHS51, *L. plantarum* LUHS135, *P. acidilactici* LUHS29, *L. plantarum* LUHS122, *L. coryniformins* LUHS71, *L. paracasei* LUHS244, *P. pentosaceus* LUHS183 and *L. faraginis* LUHS206 strains were selected for milk permeate fermentation according to their carbohydrate fermentation, antimicrobial and antifungal characteristics [23]. Pure LAB strains were stored at −80 °C in a Microbank system (Pro-Lab Diagnostics, Merseyside, UK) and grown in the Man, Rogosa and Sharpe (MRS) broth (CM 0359, Oxoid, Hampshire, UK) under anaerobic conditions, at 30 °C for 48 h prior to use. Anaerobic conditions were attained by incubation the *Petri* dishes in anaerobic jars (Oxoid, Basingstoke, Hampshire, UK), with GasPak Plus™ (BBL, Cockeysville, MD, USA).

Milk permeate was obtained from agricultural cooperative “Pienas LT” (Biruliskes, Lithuania), and stored at −18 °C before use. Furthermore, apple (variety ‘Auksis’) by-products were received from the Institute of Horticulture, Lithuanian Research Centre for Agriculture and Forestry (Babtai, Kaunas distr., Lithuania) in 2019. The whole press cake of apple by-products from juice production were composed seeds, peel and lignocellulose components. After reception in the lab, the apple by-products, obtained after juice preparation, were immediately vacuum dried in a vacuum dryer XF020 (France-Etuves, Chelles, France). Vacuum drying was performed at 45.0 ± 2.0 °C and under a pressure of 6.0 × 10^−3^ mPa. Dried samples were ground in an ultra-centrifugal mill ZM 200 (Retsch, Haan, Germany) mounted with a stainless steel sieve of 0.75 mm-Φ, and stored at room temperature (22 ± 2 °C) in light- and air-protective bags.

### 2.2. Fermentation of Milk Permeate (MP)

The *L. uvarum* LUHS245, *L. casei* LUHS210, *L. curvatus* LUHS51, *L. plantarum* LUHS135, *P. acidilactici* LUHS29, *L. plantarum* LUHS122, *L. coryniformins* LUHS71, *L. paracasei* LUHS244, *P. pentosaceus* LUHS183 and *L. faraginis* LUHS206 were incubated and multiplied in MRS broth culture medium (Biolife, Milano, Italy) at 30 °C under anaerobic conditions. A total of 3% (*v*_innoculum_/*v*_milk permeate_) of LAB with a cell concentration of 9.2 log_10_ CFU/mL (LAB count determined by method described in Section 2.4) were inoculated in milk permeate, followed by anaerobic fermentation in a modified atmosphere of carbon dioxide in a chamber incubator (Memmert GmbH + Co. KG, Schwabach, Germany) for 48 h at 30 °C.

After fermentation, different amounts of dried apple by-products in fermented milk permeate-based beverage prototypes were tested and the most favorable formulation found (according to results of the overall acceptability scores) was selected, chiefly 8% (*w*_dried apple by-products_/*w*_fermented beverages_) of apple by-products. Evaluation of the overall acceptability is described in Section 3.5.

Finally, 10 samples of MP fermented with each of the previous 10 selected LAB strains were prepared without (MP) and with (AppMP) the addition of 8% (*w/w*) apple by-products [App—with 8% of apple by-products] giving rise to a total of 20 (10 + 10) samples, *viz*.: LUHS245—fermented with LUHS245 (*L. uvarum*); LUHS210—fermented with LUHS210 (*L. casei*); LUHS51—fermented with LUHS51 (*L. curvatus*); LUHS135—fermented with LUHS135 (*L. plantarum*); LUHS29—fermented with LUHS29 (*P. acidilactici*); LUHS122—fermented with LUHS122 (*L. plantarum*); LUHS71—fermented with LUHS71 (*L. coryniformins*); LUHS244—fermented with LUHS244 (*L. paracasei*); LUHS183—fermented with LUHS183 (*P. pentosaceus*); and LUHS206—fermented with LUHS206 (*L. faraginis*). Non-fermented MP samples were tested as control (NF—non-fermented).

### 2.3. Determination of Acidity Parameters in the Beverages Throughout Fermentation Time

The acidity parameters pH and Total Titratable Acidity (TTA) of the beverages were determined throughout fermentation time (0–48 h), where 0 h corresponds to the non-fermented MP and 48 h to the final fermented beverage. The pH values of the beverages were measured and recorded using a pH electrode (PP—15, Sartorius, Goettingen, Germany). Total titratable acidity (TTA) was determined in a 10 mL of sample homogenized with 90 mL of distilled water, titrated with a volume (mL) of 0.1 mol/L NaOH to obtain a final pH value of 8.2, and expressed as Neiman degrees (°N). The measurements were undertaken in triplicate and the average values and standard deviations (SDTV’s) calculated.

### 2.4. Determination of Lactic Acid Bacteria (LAB) Viable Counts in the Beverages throughout Fermentation Time

For the evaluation of LAB viable counts throughout fermentation time (0–48 h), where 0 h corresponds to the non-fermented MP and 48 h to the final fermented beverage, 10 mL of the beverages were homogenized with 90 mL of aqueous saline (9 g/L) solution. Serial decimal dilutions of 10^−4^ to 10^−8^ with the same saline solution were prepared for inoculation. Sterile MRS agar (CM0361, Oxoid) of 5 mm thickness was used for bacterial inoculation and growth onto *Petri* dishes. The *Petri* dishes were separately inoculated with the sample suspension using the spread plate technique and were incubated under anaerobic conditions at 30 °C for 72 h. All results were expressed in log_10_ CFU/mL, taking into account the dilution factor and the amount of sample, and the average values and STDV’s of three analytical determinations were calculated. In microbial viable counts, the logarithmic transformation is required for stabilization of variance and normalization of residuals.

### 2.5. Determination of Lactose and Galactooligosaccharide (GOS) Concentration in the Initial Non-Fermented Milk Permeate (MP) and/or Final Fermented Beverages

Lactose was determined in the initial non-fermented MP (0 h) and final fermented beverages (48 h) by normal-phase (NP) high-performance liquid chromatography (HPLC) with evaporative light-scattering detection (ELSD) (NP-HPLC-ELSD). To that purpose, 0.5–1.0 g of the final beverages were diluted with approximately 70 mL of deionized water, heated to 60 °C in a water-bath for 15 min, clarified with 2.5 mL Carrez I {85 mM K_4_[Fe(CN)_6_]∙3H_2_O} and 2.5 mL Carrez II (250 mM ZnSO_4_∙7H_2_O) solutions, and made up to 100 mL with distilled/deionized water. After 15 min, the samples were filtered through a 90 mm-Φ Whatman No. 2 filter paper of 8 µm-pore size (Maidstone, UK) and were further filtered through a 25 mm-Φ disposable syringe filters of 0.22 µm-pore size (Chromafil^®^ PET-45/25 Polyester, Macherey-nagel, Dueren, Germany) before analysis. A sugar standard mixture was prepared by dissolving 0.2 g each of fructose (Sigma-Aldrich, Germany), glucose (Sigma-Aldrich, Germany), sucrose (Sigma-Aldrich, Germany) and lactose (Sigma-Aldrich, Germany) in 100 mL of deionized water. A 2 mg/mL standard sugar mixture and several decimal dilutions were prepared with deionized water, so the chromatographic quantification could be performed after separation and identification of the peaks. Test samples were diluted to fit the linear zone of the calibration curve.

An LC 1090 Hewlett Packard HPLC system (Agilent Technologies, Santa Clara, CA, USA) was employed for separation was employed. The HPLC conditions were as follows: the mobile elution system consisted in acetonitrile:deionized water (ACN:H_2_O) (75:25, *v*/*v*), a flow-rate of 1.2 mL/min, and an injection volume of 20 μL. The chromatographic separation was undertaken in an YMC-Pack Polyamine II 250 length × 4.6 mm I.D. (internal diameter), 5 μm-particle size (YMC Co., Ltd., Japan) column mounted in series with a guard holder (guard cartridge with the same characteristics of the column, and with 10 mm length × 4.6 mm I.D., 5 μm-particle size). The column temperature was set at 28.0 °C for the chromatographic separation. The detection was performed using an Evaporative Light-Scattering Detector (ELSD) LTII (Shimadzu Scientific Instruments Incorporated, Kyoto, Japan). The ELSD temperature was kept at 55 °C and 600 V. The nebulizer gas (purity of 99.9%) was air compressed at 1 bar. The photomultiplier sensitivity was adjusted to a gain of 6.

In turn, the elution, separation and quantification of GOS (galactobiose, galactotriose and galactotetraose) was determined in the final fermented beverages (48 h) and performed by normal-phase High-Performance Anion-Exchange Chromatography (NP-HPAEC) with Pulsed Amperometric Detection (PAD) (NP-HPAEC-PAD) method as described by Courtin et al. [25] with some modifications. For this purpose, a Varian Pro Star HPLC system (Varian Inc., Paolo Alto, CA., USA), equipped with two solvent delivery modules (Varian Pro Star 210), auto-sampler (Varian Pro Star 410), a column oven and pulsed amperometric detector Decade II SCC (Antek Leyden bv, Zoeterwoude, The Netherlands) with Flexcell (Antek Leyden bv) equipped with 3 mm gold (Au) working electrode, was used.

As standard references, 1.4-β-d-Galactobiose (Megazyme Ltd., Wicklow, Ireland), 3a, 4b-Galactotriose (Santa Cruz Biotechnology Inc., Santa Cruz, CA., USA) and 3α, 4β, 3α-Galactotetraose (Santa Cruz Biotechnology Inc) were used [25]. Furthermore, the GOS in the solutions of the prototypes of beverages were separated in a column CarboPac PA100, 50 mm length × 4 mm I.D. and 5 μm-particle size (Thermo Fisher Scientific, Waltham, MA., USA), mounted in series with a guard column CarboPac PA100 (Thermo Fisher Scientific, 5 mm length × 4 mm I.D., 5 μm-particle size, both preheated at 30 °C. An aliquot of 5 µL of solution was injected onto the column and eluted at a flow-rate of 1.0 mL/min according to the gradient program described by Courtin et al. [25].

The following measurement potentials (E) and time periods (t) were selected: E_1_ = +0.05 V, t_1_ = 400 ms; E_2_ = +0.75 V, t2 = 200 ms; and E_3_ = −0.40 V, t_3_ = 400 ms. All flow-lines of HPLC system and the injection cell (Flexcell) were passivated before connecting the column by flushing at 1.0 mL/min with the following sequence: ethanol (10 min), deionized water (5 min), nitric acid 1N (HNO_3_) (10 min), deionized water (5 min), 0.1% ethylenediamine tetraacetic acid disodium salt (EDTA-2Na) solution (30 min) and, finally, deionized water (30 min).

Each sample was analyzed by HPLC and HPAEC in triplicate and the average values and standard deviations (SDTV’s) determined. Results were expressed g_lactose_/100g_sample_ and mg_GOS_/100mL_sample_.

### 2.6. Evaluation of the Antimicrobial Activity in the Milk Permeate (MP) and Final Fermented Beverages by the Agar Well-Diffusion and Liquid Culture Medium Methods

All the initial non-fermented MP (0 h) and final fermented beverages (48 h) were assessed for their antimicrobial activities against a variety of 15 pathogenic and opportunistic bacterial strains (*Klebsiella pneumoniae*, *Salmonella enterica* 24 SPn06, *Pseudomonas aeruginosa* 17–331, *Acinetobacter baumanni* 17–380, *Proteus mirabilis*, methicillin-resistant *Staphylococcus aureus* (MRSA) M87fox, *Enterococcus faecalis* 86, *Enterococcus faecium* 103, *Bacillus cereus* 18 01, *Streptococcus mutans*, *Enterobacter cloacae*, *Citrobacter freundii*, *Staphylococcus epidermis*, *Staphylococcus haemolyticus* and *Pasteurella multocida*) by the agar well diffusion method and in liquid medium.

For the agar well diffusion assay, suspensions of 0.5 McFarland standard of each pathogenic bacteria strains were inoculated by spread plate onto the surface of Mueller–Hinton (MD) agar (Oxoid, Basingstoke, UK) *Petri* dishes using sterile cotton swabs as described by Bartkiene et al., 2012 [4] and 2020 [24]. Wells of 6 mm diameter were punched in the agar and filled with 50 µL of the final prepared beverages and control. The antimicrobial activities against the tested bacteria were established by measuring the inhibition zone diameters (mm). The experiments were made in triplicate and the average and standard deviation of the inhibition zones was calculated.

To evaluate the antimicrobial activity of beverages in liquid culture medium, the final prototypes were diluted in a 1:3 (*v*/*v*) ratio with physiological (sodium chloride) solution. Then, to the 0.5 mL of the diluted beverages, 0.1 mL of the pathogenic and opportunistic bacterial strains, grown in a selective medium, was added and incubated at 35 °C for 24 h. After incubation, the viable pathogenic and opportunistic bacterial strains in beverage prototypes were assessed by plating them on selective culture medium. The results were interpreted as negative or positive if the pathogens did not grow or grow on the selective culture medium, respectively. Experiments were performed in triplicate.

### 2.7. Evaluation of the Overall Acceptability and Emotions Induced by the Milk Permeate (MP) and Final Fermented Beverages

The overall acceptability of all the initial non-fermented MP (0 h) and fermented beverages (48 h) was determined by 50 judges, according to the International Standards Organization method 8586-1 [26], using a 10-point scale ranging from 0 (extremely dislike) to 10 (extremely like).

**Figure 1:**
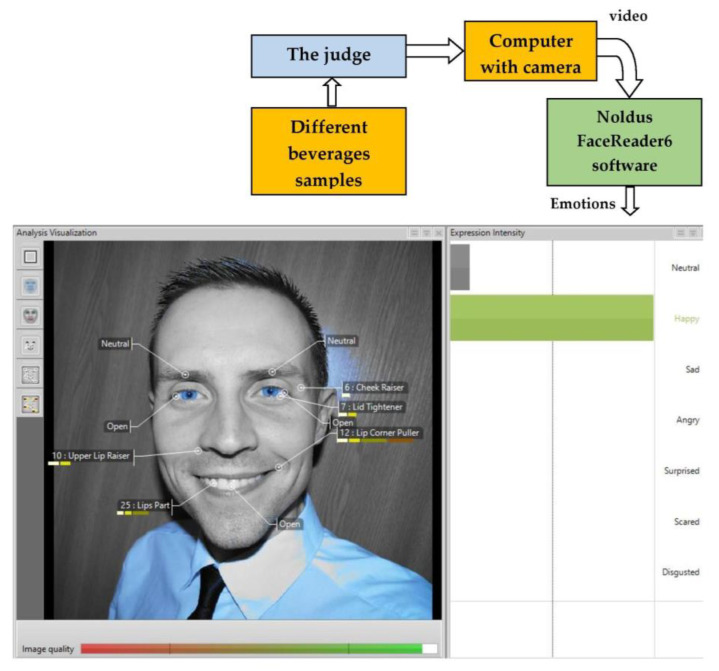
Analysis of the emotions induced by the final beverages using the FaceReader 6 software (Noldus Information Technology, Wageningen, The Netherlands), and further scoring the 8 emotion patterns: neutral, happy, sad, angry, surprised, scared, disgusted and contempt [25,26].

The beverage prototypes were also tested by applying FaceReader 6.0 software (Noldus Information Technology, Wageningen, The Netherlands) (Figure 1), with a scoring scale of 8 emotion patterns (neutral, happy, sad, angry, surprised, scared, disgusted, contempt) according to Bartkiene et al. (2019) [27]. The whole procedure was filmed using a Microsoft LifeCam Studio webcam mounted on a laptop facing the participants, and Media Recorder (Noldus Information Technology) software. Special care was taken to ensure good illumination of participant faces. The recordings, using a resolution of (1280 × 720) pixels at 30 frames per second, were saved as AVI files and analyzed frame by frame with FaceReader 6 software, scaling the eight basic emotion patterns to one (maximum intensity of the fitted model). In addition, the FaceReader also analyzed the valence, which indicates whether the person’s emotional status is positive or negative. ‘Happy’ was the only positive emotion, while ‘Sad’, ‘Angry’, ‘Scared’ and ‘Disgusted’ were considered to be negative emotions. ‘Surprised’ can be either positive or negative.

The valence was calculated as the intensity of ‘Happy’ minus the intensity of the negative emotion with the highest intensity. Valence scores ranged from −1 and 1. For each sample, the section of intentional facial expression (from the exact point at which the subject had finished raising their hand to give the signal until the subject started lowering their hand again) was extracted and used for statistical analysis. The FaceReader contained an image quality bar (Figure 1), which gave a good indication of how well the program was able to model the face depicted in the image. For the best image quality, the main attention was focused on camera position and illumination. For this reason, participants were asked to sit and look directly into the camera. For statistical analysis, the maximum values of facial expression patterns of the respective sections were used.

### 2.8. Statistical Analysis

All analysis was performed at least in triplicate (*n* = 3) and were expressed as the average ± standard deviation. Results were analyzed via one-way analysis of variance (1-way ANOVA) using statistical package SPSS for Windows XP version 15.0 (SPSS Inc., Chicago, IL, USA, 2007). The factors considered independently were the fermentation time (*n* = 4, *t* = 0, 6, 12, 24, 48 h) and type of formulations (*n* = 21) in the final fermented beverages and the dependent variables are summarized in Table 1. When F-test were significant, Tukey-HSD (HSD—honestly significant difference) post-hoc tests (with correction to control for type-I error) were applied. Prior to the 1-way ANOVA, the homogeneity of variance was verified using the Shapiro test, whereas the normality was verified using the Levene’s test. An *α*-value of 0.05 was used thus whenever *p*-values were equal or below to 0.05 (*p* < 0.05), the means were considered statistically different.

## 3. Results and Discussion

### 3.1. Determination of Acidity Parameters in the Beverages throughout Fermentation Time

Data presented in Table 1 summarize the acidity parameters (pH and TTA) of the final fermented beverages throughout 48 h of fermentation (0–48 h) with different LAB strains. One figured out that the pH values decreased significantly (*p* ≤ 0.05) on average by 25.2 and 32.6% in 24 and 48 h fermented beverages, respectively (except in 24 h fermented MPLUHS210), compared to the control (non-fermented milk permeate, MPNF). The lowest pH value of MP (on average by 35.4%) was determined in 48 h fermented MPLUHS183, compared to MPNF. However, significant differences between pH values of all fermented samples with the same fermentation time were not found.

Fermented MP samples showed a significant (*p* ≤ 0.05) higher TTA (on average by 1.2-fold) after 24 and 48 h of fermentation, compared to MPNF. The highest TTA (11.0°N) was found for the sample MPLUHS183 after 48 h of fermentation. As expected, there was a strong negative correlation in the tested samples between pH and TTA (*r* = −0.85).

The pH values of commercial non-alcoholic, non-dairy beverages range from 2.1 (lime juice concentrate) to 7.4 (spring water). The presence of organic acids contributes substantially to the flavor profile, giving to the beverage a distinctive taste and providing a tartness and tangy taste that helps to balance the sweetness of sugar present in the beverages. Indeed, they are key-factors towards the taste and aroma of beverages [28,29]. Our results revealed that the acidity values of fermented beverages prepared with different LAB strains for fermentation gradually increased in the course of fermentation, as a result of the LAB metabolism of carbohydrates presented therein, and these findings are in agreement with other authors [30]. As a matter of fact, the main sugar in milk permeate was lactose, which was fermented by LAB. These results are in agreement with those of Atallah (2015) [31] and Gholami et al. (2012) [32]. Gholami et al. (2012) [32] stated that during the fermentation of MP, the LAB starter cultures showed post-acidification, thus with the concomitant reduction of pH and increase of TTA.

### 3.2. Determination of Lactic Acid Bacteria (LAB) Viable Counts in the Beverages throughout Fermentation Time

LAB viable counts in final fermented functional beverages after 48 h of fermentation are tabulated in Table 1. According to these results, non-fermented MP did not contain LAB, that is the LAB viable counts at the end of fermentation in each prototype of beverage come from the initial controlled inoculation with each single starter culture.

The LAB viable counts increased throughout fermentation time, and ranging from 6.34 to 8.82 log_10_(CFU/mL) in fermented MP. The highest LAB viable cells count was found in MPLUHS210 after 48 h of fermentation. However, every LAB strain had demonstrated any significant influence on LAB growth in fermented samples with the same fermentation time. As predicted, a very strong negative correlation between the samples pH and LAB viable counts was found (*r* = −0.90), as well as a very strong positive correlation between the samples.

Correlation between TTA and LAB viable count was verified (*r* = 0.95), meaning that LAB had a major role on the production of metabolites (mainly organic acids) with high impact on the acidification of the beverages (and also in the taste and antimicrobial activity). Milk permeate, as a microbial growth medium, can be used by a wide range of bacteria to yield processed permeate products [33]. The increase in bacterial viable counts is related with their specific ability to grow and multiply in such bulk growth medium as well as with their specific tolerance to the significant increase of the acidity during fermentation [24]. Accumulation of lactic acid, which is produced during lactose fermentation, could influence LAB physiology, metabolism and elicit acid stress [34]. LAB resistance to acidic conditions is related to the regulation of fundamental metabolic pathways, proton pump, changes of cell membrane composition and other mechanisms [35]. Our results disclosed that all selected LAB grew well in MP medium and kept their viability even at low pH values. Similar trends were published by Angmo et al. (2016) [36] and Aamer et al. (2017) [37]. The acid tolerance ability of LAB is known in many other fermentation processes [6]. Besides, when co-cultured LAB starter cultures are employed in fermentation processes, it is of first importance to select compatible strains, since their antagonistic and synergistic interactions are based on the metabolism of carbohydrates and amino acids [6,12,13,15,16,17].

### 3.3. Determination of Lactose and Galactooligosaccharide (GOS) Concentration in the Initial Non-Fermented Milk Permeate (MP) and/or Final Fermented Beverages

**Figure 2:**
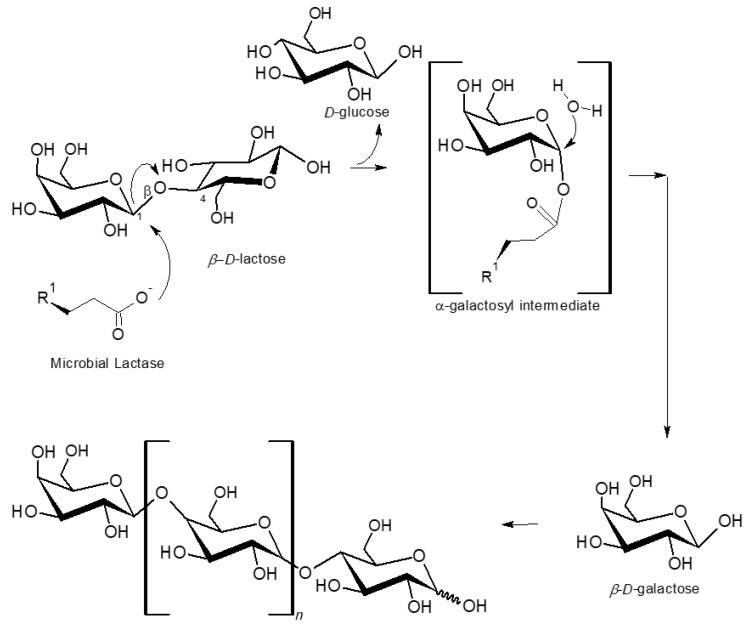
Structural chemical formula of β-d-lactose and its hydrolysis by a microbial lactase (β-galactosidase) in their monomers d-glucose and β-d-galactose, and the formation of β-d-galactose derivative galactooligosaccharides (GOSs) during the microbial enzymatic hydrolyses of lactose. Adapted from Rocha and Guerra (2020) [2].

**Figure 3:**
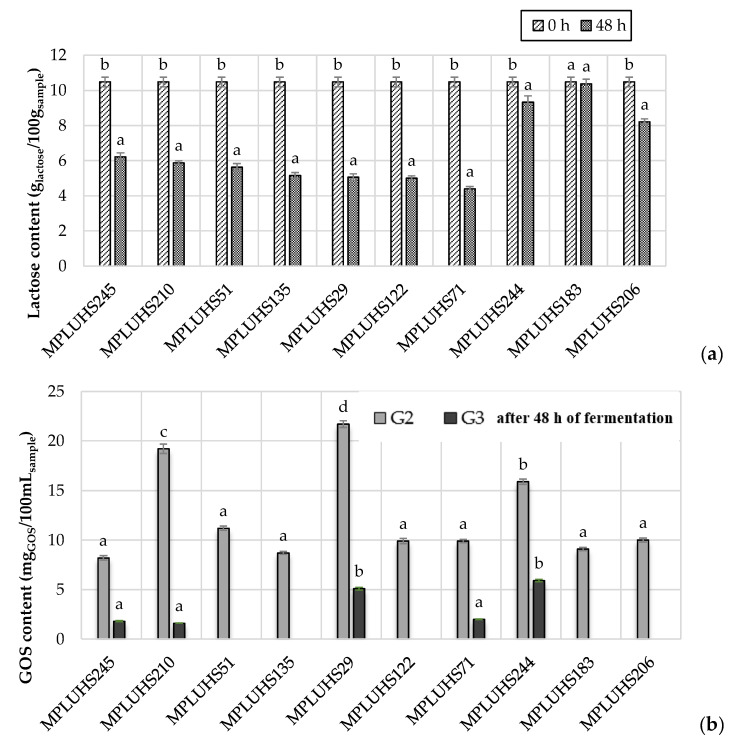
Average values and standard deviations (Mean ± STDV) (*n* = 3) of the lactose (g_lactose_/100 g_sample_) (**a**) and galactooligosaccharides (GOS) (mg_GOS_/100 mL_sample_) (**b**) content in the milk permeate (MP) (0 h) and/or final fermented beverages (48 h) without the addition of apple by-products. MP—milk permeate; MP_LUHS245_—fermented with LUHS245 (*L. uvarum)*; MP_LUHS210_—fermented with LUHS210 (*L. casei*); MP_LUHS51_—fermented with LUHS51 (*L. curvatus*); MP_LUHS135_—fermented with LUHS135 (*L. plantarum)*; MP_LUHS29_—fermented with LUHS29 (*P. acidilactici)*; MP_LUHS122_—fermented with LUHS122 (*L. plantarum)*; MP_LUHS71_—fermented with LUHS71 (*L. coryniformins)*; MP_LUHS244_—fermented with LUHS244 (*L. paracasei)*; MP_LUHS183_—fermented with LUHS183 (*P. pentosaceus)*; MP_LUHS206_—fermented with LUHS206 (*L. faraginis)*; G2—galactobiose; G3—galactotriose. Supercripts ^a–d^—Mean values with different letters are significantly different (*p* ≤ 0.05) for the same type of galactooligosaccharide (**b**).

Figure 2 shows the general metabolic pathway for the microbial enzymatic hydrolysis of the disaccharide β-D-lactose (also known as milk sugar) into the monomers D-glucose and β-d-galactose, and the subsequent formation of galactooligosaccharides (GOSs) (or simply oligosaccharides) with different number of β-d-galactose monomers [3]. In the current study three GOSs were analyzed, *viz*. galactobiose (dimer, G2), galactotriose (trimer, G3) and galactotetraose (tetramer, G4) (Figure 3). GOS production depends on the nature of the microbial enzymes. As referred above, GOSs are a class of carbohydrates recognized as functional compounds, possessing prebiotic properties, thus with high commercial interest in the agro-food (e.g., dairy, infant food and animal and fish feed) and nutraceutical industries. GOS are not digested in the small intestine and when reaching the colon, they stimulate the growth of lactose-positive LAB. GOS exhibit several health promoting traits and effects. As examples are: they may increase the absorption of calcium and magnesium; they are low in calories and exhibit bacteriostatic and non-carcinogenic properties; and the growth of LAB in the gastrointestinal tract may be of first importance in infants and, particularly, in non-breastfed infants [3].

The lactose and GOS concentrations in the initial milk permeate (0 h) (MP) and its final fermented beverages (48 h) (without the addition of apple by-products, inoculated with different LAB strains are given in Figure 3. In comparison with non-fermented MP (0 h), the total content of lactose in the final fermented beverages (48 h) decreased significantly (*p* ≤ 0.05) on average by 37.7% after 48 h of fermentation, except for MPLUHS183, showing the ability of these LAB strains to metabolize lactose. In comparison with the MPNF, the highest decrease of lactose content (by 58.1%) was found in the fermented MPLUHS71.

LAB strains had a significant influence (*p* ≤ 0.05) on GOS synthesis in fermented MP—thus highlighting the importance of LAB fermentation of milk permeate-based by-products, due to the ability to metabolize lactose and the concomitant production of metabolites with antimicrobial and antioxidant properties, as well as to the fact of being beneficial microorganisms and their metabolites are classified as Generally Regarded as Safe (GRAS) and Qualified Presumption of Safety (QPS) [10]. The highest total content of GOS (26.80 mg_GOS_/100 mL_sample_) was obtained in fermented MPLUHS29, while the lowest concentration (8.7 mg_GOS_/100 mL_sample_) was noticed in fermented MPLUHS135. Galactobiose (G2) in combination with galactotriose (G3) were formed in MPLUHS245, MPLUHS210, MPLUHS29, MPLUHS71 and MPLUHS244. In the remaining samples only G2 was synthesized. Galactotetraose (G4) in fermented MP samples were not detected at all.

In this study, only weak or very weak correlations between GOS content and pH, TTA, LAB viable counts or initial lactose content were obtained (*r* = 0.22, *r* = −0.15, *r* = −0.09, *r* = −0.04, respectively). GOS are produced through transgalactosylation reaction and are characterized in terms of their prebiotic, immunomodulation and functional properties in foods [38]. The changes in GOS yield are closely related with enzyme origin, lactose concentration and GOS itself, because they can be further hydrolyzed to the monomers [39]. This is likely to be the reason to not having been found a high positive correlation between GOS and TTA, LAB and the initial lactose content, and a high negative correlation between GOS and pH. The ability of *Lactobacillus* spp., *P. pentosaceus* and *P. acidilactici* to synthesized GOS in fermented dairy media was reported by other authors [40,41,42,43]. However, observed differences between results in these studies are highly influenced by the type of microorganisms used and the initial substrate composition. The enzymatic activity of different LAB strains used for this experiment could influence the variety and yield of GOS, as well as the degree of lactose hydrolysis. Anyway, the formation of GOS in MP led undoubtedly to the improved functional value of fermented beverages.

### 3.4. Evaluation of the Antimicrobial Activity in the Milk Permeate (MP) and Final Fermented Beverages by the Agar Well-Diffusion and Liquid Culture Medium Methods

The antimicrobial activities of the final fermented beverage prototypes with apple by-products (AppMP) and without (MP) against a variety of pathogenic and opportunistic bacterial strains are given in Table 2 and Table 3, by measuring the diameter of inhibition zones (DIZ) and growth detection in liquid medium, respectively. Both types of the final fermented beverage prototypes (MP and AppMP) did not possess antimicrobial activities against *Klebsiella pneumoniae*, *Acinetobacter baumanni* 17–380, *Proteus mirabilis*, *Citrobacter freundii*, *Enterococcus faecalis* 86 and *Enterococcus faecium* 103. All MP beverages inhibited the growth of *Streptococcus mutans* (DIZ ranged from 10.4 ± 0.3 to 15.9 ± 0.4 mm), *Pasteurella multocida* (DIZ ranged from 14.3 ± 0.3 to 23.3 ± 0.3 mm) and *Streptococcus epidermis* (except MPLUHS135 and MPLUHS29). Additionally, in MP the antimicrobial activity of MPLUHS210 against *Salmonella enterica*, *Pseudomonas aeruginosa* 17–331, *Bacillus cereus*, *Enterobacter cloacae* and *Staphylococcus haemolyticus* were observed. The latter bacteria strain was also inhibited by MPLUHS135 and MPLUHS51.

It must be emphasized that a broader inhibition spectrum of the samples prepared with fermented MP with apple by-products (AppMP) was unfolded. All of AppMP beverages inhibited *Pasteurella multocida*. Comparing AppMP beverages, *Pseudomonas aeruginosa* 17–331 was inhibited by 4 samples of a total of 11 analyzed: AppMPLUHS122 (DIZ 12.0 ± 0.4 mm), AppMPLUHS244 (DIZ 12.3 ± 0.5 mm), AppMPLUHS183 (DIZ 13.4 ± 0.6 mm) and AppMPLUHS206 (DIZ 11.6 ± 0.2 mm). The methicillin-resistant *Staphylococcus aureus* MRSA was inhibited by AppMPLUHS245 (DIZ 11.0 ± 0.7 mm), AppMPLUHS210 (DIZ 12.06 ± 0.3 mm), AppMPLUHS122 (DIZ 11.03 ± 0.2 mm), and AppMPLUHS71 (DIZ 12.4 ± 0.5 mm). *Bacillus cereus* 18 01 was inhibited by AppMPLUHS210 AppMPLUHS122, AppMPLUHS71, AppMPLUHS244, AppMPLUHS183 and AppMPLUHS206 (average DIZ of 11.6 ± 0.29 mm). *Streptococcus epidermis* was inhibited by AppMPLUHS245, AppMPLUHS210, AppMPLUHS51, AppMPLUHS135 and AppMPLUHS29 (DIZ ranged from 11.2 ± 0.1 to 18.9 ± 0.6 mm). It should also be mentioned that *Streptococcus mutans* was inhibited by AppMPLUHS210, AppMPLUHS29 and AppMPNF (DIZ of 17.4 ± 0.3, 20.1 ± 0.4 and 14.3 ± 0.6 mm, respectively).

The antimicrobial activities of the tested beverage prototypes against pathogenic and opportunistic bacteria in liquid medium (Table 3) revealed that the MPLUHS245, MPLUHS210 and AppMPLUHS71 inhibited the highest number of those microorganism—13 of the 15. MPLUHS135 inhibited 6 of the 15 tested pathogens and this was the lowest antimicrobial activity between all tested beverages. In general, MP and AppMP fermented beverages inhibited similar number of pathogens (on average by 10 of the 15 tested pathogens).

LAB have been used in food processing because of their capability to increase the healthiness and sensory properties of food [44]. However, the microbial safety of food products could be improved or ensured by LAB antimicrobial compounds released to the media, such as organic acids, bacteriocins, hydrogen peroxide, diacyls, etc. [6,9,10,11,12,13,15,16,17,45]. The antimicrobial activity of LAB strains belonging to *Lactobacillus* is mainly related with production of organics acids, including lactic and acetic acids [46]. These acids possess a wider range of antimicrobial activity, while bacteriocins basically suppress the growth of Gram-positive bacteria [12,13,15,16,17]. The antimicrobial activity of the LAB strains used in this experiment were also examined in our previous research [4].

The increasing number of research studies have found that apples can possess some antimicrobial activity. Conventionally produced apples inhibited *Bacillus cereus*, while the organic peel demonstrated antimicrobial activity against *Escherichia coli* 0157:07 [47]. It was also reported that the presence of phloretin and its glycosylated derivatives in apples are able to inhibit growth of Gram-positive bacteria, and are active against some strains of the Gram-negative bacteria [48]. In the research of Radenkovs (2018) [49] wild apple pomace oil showed inhibitory capacity against both Gram-positive and Gram-negative bacteria strains. These could explain the antimicrobial activity of AppMPNF against *Streptococcus epidermis* and *Streptococcus mutans* found din our research data. However, Liya and Siddique (2018) [50] reported that methanolic and ethanolic extracts of green apple did not show any inhibition of tested pathogens.

### 3.5. Evaluation of the Overall Acceptability and Emotions Induced by the Milk Permeate (MP) and Final Fermented Beverages

The results of overall acceptability (OA) and emotions induced by the final fermented beverage prototypes are given in Table 4. The addition of apple by-products (App) to fermented MP increased the OA of AppMP beverages by 26.8%, when compared to beverages without App. AppMPLUHS183 beverage was the most acceptable (score of 7.1) by the judge panel, while non-fermented MP was selected as the least liked sample. Significant differences in OA (*p* < 0.05) among most of the fermented MP beverages (except MPLUHS245, MPLUHS244 and MPLUHS206) were found when compared to the control MPNF. Likewise, in comparison with AppMPNF almost all AppMP beverages (except MPLUHS135 and AppMPLUHS245) were significantly different in terms of OA.

As could be perceived from our results, the fermentation with different LAB and addition of apple by-products improved the sensory perception of the final fermented beverages and increased the acceptability. The positive changes in OA could be explained on the basis of the slight variation in the composition of the fermented products. Moreover, a higher level of the flavor compounds from apple by-products provided a more expressive taste of fermented drinks that the sensory analysis panel enjoyed better. A negative moderate (*r* = −0.53) and a strong positive (*r* = 0.72 and *r* = 0.73) correlations between AppMP acceptability and pH, TTA or lactose content, respectively, were obtained in this study—which shows a trend for acceptance of higher contents of acidity and lactose. Similar trends were reported by Pereira and Rodrigues (2018) [51], Janiaski et al. (2016) [52], and Balthazar et al. (2018) [53].

The analysis of facial expressions with FaceReader software showed a diverse distribution of emotions between each tested beverage. Besides “neutral”, which is usually expressed as the most intensive facial expression, “happy”, “sad” and “angry” were elicited more intensely than the rest during this research. Significant differences (*p* ≤ 0.05) in “happy” emotion among the tested beverages were observed. MPLUHS71, MPLUHS24, MPLUHS183, MPLUHS206 and MPNF intensity scores of “happy” were significantly higher when compared to the other samples. However, it was noticed that other emotions showed ambiguous results, because not all of them were statistically reliable. It is important to mention that a negative moderate (*r* = −0.54) and positive weak (*r* = 0.3) correlations between beverages acceptability and emotions “happy” or “angry” were respectively encountered. Examination of emotional response to food products could provide additional information about consumers’ perception of food and acceptability of new products [54]. In this study the obtained results are in agreement with those given by Juodeikiene et al. (2014) [54], Leitch et al. (2015) [55] and Danner et al. (2014) [56].

## 4. Conclusions

The functional beverages are a healthy alternative in human nutrition, whereas their production can simultaneously lead to the sustainable valorization of dairy industry by-products. The results of this study showed that fermented MP beverage prototypes with selected LAB strains contained GOS (from 8.7 to 26.8 mg_GOS_/100 mL_sample_) and possessed important antimicrobial activity against most of the tested bacterial pathogens. Fermented MP showed a significantly (*p* ≤ 0.05) lower pH and higher TTA compared to non-fermented MP. The type of LAB strains had a significant (*p* ≤ 0.05) influence on GOS synthesis in fermented MP. The highest total content of GOS (galactobiose and galactotriose) was found in fermented MPLUHS29. The antimicrobial activity of all MP beverages against *Streptococcus mutans* and *Pasteurella multocida* was observed, and the latter one was also inhibited by all AppMP beverages. MPLUHS245, MPLUHS210 and AppMPLUHS71 beverages inhibited the highest number of tested pathogens, chiefly 13 of the 15. The addition of apple by-products in fermented MP increased the overall acceptability of the final fermented beverages (by 26.8% in comparison with beverages without App). AppMPLUHS183 was the most acceptable beverage. The diverse distribution of emotions between each tested beverage was noticed. MPLUHS71, MPLUHS24, MPLUHS183 and MPLUHS206 intensity scores of “happy” were significantly higher compared to other samples. This research study highlights the real potential of the bioactive prototypes of MP-based fermented beverages (with and without the addition of apple juice by-products), in the commercial point of view, as well as in the promotion of healthy eating habits and a circular economy.

## Figures and Tables

**Table 1:** Average values and standard deviations (Mean ± STDV) (*n* = 3) of the acidity parameters, pH and total titratable acidity (TTA), and total lactic acid bacteria (LAB) viable counts in the milk permeate (MP) samples throughout fermentation (t_sampling_ = 0, 6, 12, 24, 48 h).

Milk Permeate Samples	Duration of Fermentation														
	0 h			6 h			12 h			24 h			48 h		
	pH	TTA (ºN)	LAB Viable Counts [(log_10_(CFU/mL)]	pH	TTA (ºN)	LAB Viable Counts [(log_10_(CFU/mL)]	pH	TTA (ºN)	LAB Viable Counts [(log_10_(CFU/mL)]	pH	TTA (ºN)	LAB Viable Counts [(log_10_(CFU/mL)]	pH	TTA (ºN)	LAB Viable Counts [(log_10_(CFU/mL)]
MPNF	5.88 ± 0.8 ^a^	3.0 ± 0.1 ^a^	nd	5.88 ± 0.8 ^a^	3.0 ± 0.14 ^a^	nd	5.88 ± 0.8 ^b^	3.0 ± 0.14 ^a^	nd	5.88 ± 0.8 ^b^	3.0 ± 0.14 ^a^	nd	5.88 ± 0.8 ^b^	3.0 ± 0.14 ^a^	nd
MP_LUHS245_	5.58 ± 0.11 ^a^	3.6 ± 0.1 ^a^	6.85 ± 0.23 ^a^	5.57 ± 0.17 ^a^	3.6 ± 0.13 ^a^	6.79 ± 0.18 ^a^	5.48 ± 0.2 ^b^	3.5 ± 0.09 ^a^	6.89 ± 0.25 ^a^	4.25 ± 0.09 ^a^	6.4 ± 0.24 ^c^	7.06 ± 0.18 ^a^	3.97 ± 0.07 ^a^	9.5 ± 0.29 ^b^	8.68 ± 0.39 ^a^
MP_LUHS210_	5.55 ± 0.14 ^a^	3.4 ± 0.14 ^a^	7.15 ± 0.26 ^a^	5.36 ± 0.11 ^a^	3.8 ± 0.15 ^a^	7.47 ± 0.27 ^a^	5.23 ± 0.1 ^b^	4.3 ± 0.1 ^b^	8.10 ± 0.28 ^b^	5.38 ± 0.12 ^b^	5.3 ± 0.23 ^b^	8.34 ± 0.32 ^a^	4.29 ± 0.16 ^a^	8.6 ± 0.17 ^b^	8.82 ± 0.23 ^a^
MP_LUHS51_	5.57 ± 0.3 ^a^	2.9 ± 0.09 ^a^	6.83 ± 0.16 ^a^	5.60 ± 0.24 ^a^	3.2 ± 0.1 ^a^	6.91 ± 0.21 ^a^	5.65 ± 0.19 ^b^	3.6 ± 0.1 ^a^	7.38 ± 0.26 ^a^	4.19 ± 0.13 ^a^	6.2 ± 0.37 ^c^	7.49 ± 0.11 ^a^	3.88 ± 0.25a	9.7 ± 0.41 ^b^	8.36 ± 0.28a
MP_LUHS135_	5.61 ± 0.3 ^a^	3.1 ± 0.9 ^a^	7.46 ± 0.21 ^a^	5.48 ± 0.19 ^a^	3.4 ± 0.07 ^a^	7.43 ± 0.17 ^a^	5.22 ± 0.21 ^b^	4.0 ± 0.1 ^b^	7.57 ± 0.14 ^a^	4.38 ± 0.09a	7.5 ± 0.29 ^c^	7.98 ± 0.14 ^a^	4.03 ± 0.08 ^a^	9.4 ± 0.19 ^b^	8.58 ± 0.24 ^a^
MP_LUHS29_	5.59 ± 0.15 ^a^	3.2 ± 0.03 ^a^	7.51 ± 0.26 ^a^	5.32 ± 0.27 ^a^	3.5 ± 0.07 ^a^	7.62 ± 0.21 ^a^	5.19 ± 0.13 ^b^	3.7 ± 0.07 ^a^	7.71 ± 0.28 ^a^	4.32 ± 0.11 ^a^	6.9 ± 0.26 ^c^	7.99 ± 0.22 ^a^	3.91 ± 0.23 ^a^	9.5 ± 0.19 ^b^	8.19 ± 0.23 ^a^
MP_LUHS122_	5.62 ± 0.12 ^a^	3.5 ± 0.9 ^a^	7.08 ± 0.19 ^a^	5.36 ± 0.22 ^a^	3.8 ± 0.15 ^a^	7.16 ± 0.1 ^a^	5.22 ± 0.17 ^b^	4.1 ± 0.25 ^b^	7.25 ± 0.22 ^a^	4.33 ± 0.12 ^a^	5.8 ± 0.14 ^b^	7.68 ± 0.23 ^a^	4.04 ± 0.15 ^a^	8.7 ± 0.22 ^b^	8.64 ± 0.33 ^a^
MP_LUHS71_	5.56 ± 0.17 ^a^	2.8 ± 0.12 ^a^	7.74 ± 0.15 ^a^	5.47 ± 0.14 ^a^	3.4 ± 0.13 ^a^	7.84 ± 0.19 ^a^	5.30 ± 0.19 ^b^	3.8 ± 0.08 ^a^	7.96 ± 0.29 ^a^	4.38 ± 0.12 ^a^	6.1 ± 0.12 ^c^	8.18 ± 0.31 ^a^	3.89 ± 0.12a	9.6 ± 0.16 ^b^	8.78 ± 0.25 ^a^
MP_LUHS244_	5.58 ± 0.17 ^a^	2.9 ± 0.06 ^a^	6.96 ± 0.23 ^a^	5.39 ± 0.2 ^a^	3.2 ± 0.09 ^a^	7.06 ± 0.21 ^a^	5.33 ± 0.16 ^b^	3.5 ± 0.25 ^a^	7.11 ± 0.17 ^a^	4.26 ± 0.09 ^a^	7.6 ± 0.28 ^c^	7.67 ± 0.26 ^a^	3.9 ± 0.13 ^a^	10.6 ± 0.21 ^c^	8.06 ± 0.31 ^a^
MP_LUHS183_	5.61 ± 0.11 ^a^	3.5 ± 0.2 ^a^	7.16 ± 0.19 ^a^	5.35 ± 0.1 ^a^	3.7 ± 0.10 ^a^	6.91 ± 0.26 ^a^	5.25 ± 0.13 ^b^	4.2 ± 0.21 ^b^	7.01 ± 0.27 ^a^	4.32 ± 0.09 ^a^	7.9 ± 0.15 ^c^	7.52 ± 0.27 ^a^	3.8 ± 0.15 ^a^	11.0 ± 0.35 ^c^	8.10 ± 0.22 ^a^
MP_LUHS206_	5.57 ± 0.12 ^a^	3.3 ± 0.2 ^a^	6.34 ± 0.17 ^a^	5.03 ± 0.18 ^a^	3.2 ± 0.12 ^a^	6.87 ± 0.24 ^a^	4.16 ± 0.08 ^a^	3.1 ± 0.18 ^a^	7.05 ± 0.15 ^a^	4.16 ± 0.12 ^a^	7.6 ± 0.23 ^c^	7.19 ± 0.32 ^a^	3.9 ± 0.08 ^a^	10.8 ± 0.41 ^c^	7.68 ± 0.14 ^a^

Supercripts ^a–c^—Mean values with different letters between a lines are significantly different (*p* ≤ 0.05). LAB—lactic acid bacteria; CFU—colony-forming units; TTA—total titratable acidity; nd—not detected. MP—milk permeate; MP_LUHS245_—fermented with LUHS245 (*L. uvarum)*; MP_LUHS210_—fermented with LUHS210 (*L. casei)*; MP_LUHS51_—fermented with LUHS51 (*L. curvatus)*; MP_LUHS135_—fermented with LUHS135 (*L. plantarum);* MP_LUHS29_—fermented with LUHS29 (*P. acidilactici);* MP_LUHS122_—fermented with LUHS122 (*L. plantarum)*; MP_LUHS71_—fermented with LUHS71 (*L. coryniformins)*; MP_LUHS244_—fermented with LUHS244 (*L. paracasei)*; MP_LUHS183_—fermented with LUHS183 (*P. pentosaceus)*; MP_LUHS206_—fermented with LUHS206 (*L. faraginis)*; MPNF—non-fermented.3.2. Determination of lactic acid bacteria (LAB) viable counts in the beverages throughout fermentation time.

**Table 2:** Average values and standard deviations (Mean ± STDV) (*n* = 3) of the diameter of inhibition zones (mm) in the milk permeate (MP) (0 h) and final fermented beverages (24 h), without and with addition of apple by-products, against 15 pathogenic and opportunistic bacterial strains.

Samples	Diameter of Inhibition Zones (DIZ) (mm)														
	Pathogenic and Opportunistic Bacterial Strains														
	*1*	*2*	*3*	*4*	*5*	6	*7*	*8*	*9*	*10*	*11*	*12*	*13*	*14*	*15*
*Without apple by-products*															
MP_LUHS245_	nd	nd	nd	nd	nd	nd	nd	nd	nd	15.4 ± 0.3 ^c^	nd	nd	13.0 ± 0.6 ^b^	nd	20.4 ± 0.6 ^d^
MP_LUHS210_	nd	11.0 ± 0.2	12.5 ± 0.1	nd	nd	nd	nd	nd	12.6 ± 0.7 ^c^	15.9 ± 0.4 ^c^	11.7 ± 0.8	nd	15.4 ± 0.3 ^c^	12.9 ± 0.5 ^b^	14.7 ± 0.2 ^b^
MP_LUHS51_	nd	nd	nd	nd	nd	nd	nd	nd	nd	13.2 ± 0.9 ^b^	nd	nd	15.9 ± 0.6 ^c^	12.7 ± 0.3 ^b^	14.3 ± 0.4 ^b^
MP_LUHS135_	nd	nd	nd	nd	nd	nd	nd	nd	nd	13.5 ± 0.6 ^b^	nd	nd	nd	10.2 ± 0.6 ^a^	20.2 ± 0.9 ^d^
MP_LUHS29_	nd	nd	nd	nd	nd	nd	nd	nd	nd	12.7 ± 0.4 ^b^	nd	nd	nd	nd	15.0 ± 0.1 ^b^
MP_LUHS122_	nd	nd	nd	nd	nd	nd	nd	nd	nd	12.6 ± 0.8 ^b^	nd	nd	10.7 ± 0.6 ^a^	nd	20.4 ± 0.6 ^d^
MP_LUHS71_	nd	nd	nd	nd	nd	nd	nd	nd	nd	10.4 ± 0.3 ^a^	nd	nd	11.9 ± 0.4 ^a^	nd	23.3 ± 0.3 ^e^
MP_LUHS244_	nd	nd	nd	nd	nd	nd	nd	nd	nd	15.3 ± 0.2 ^c^	nd	nd	10.2 ± 0.1 ^a^	nd	20.7 ± 0.6 ^d^
MP_LUHS183_	nd	nd	nd	nd	nd	nd	nd	nd	nd	15.4 ± 0.9 ^c^	nd	nd	10.3 ± 0.2 ^a^	nd	20.6 ± 0.5 ^d^
MP_LUHS206_	nd	nd	nd	nd	nd	nd	nd	nd	nd	14.7 ± 0.7 ^c^	nd	nd	10.6 ± 0.6 ^a^	nd	20.2 ± 0.6 ^d^
MPNF	nd	nd	nd	nd	nd	nd	nd	nd	nd	nd	nd	nd	nd	nd	nd
*With apple by-products*															
AppMP_LUHS245_	nd	nd	nd	nd	nd	11.0 ± 0.7 ^a^	nd	nd	nd	nd	nd	nd	17.3 ± 0.1 ^d^	nd	17.3 ± 0.2 ^c^
AppMP_LUHS210_	nd	nd	nd	nd	nd	12.6 ± 0.3 ^b^	nd	nd	12.3 ± 0.2	17.4 ± 0.3 ^d^	nd	nd	11.6 ± 0.2 ^a^	nd	18.6 ± 0.4 ^c^
AppMP_LUHS51_	nd	nd	nd	nd	nd	nd	nd	nd	nd	nd	nd	nd	17.2 ± 0.4 ^d^	nd	14.5 ± 0.9 ^b^
AppMP_LUHS135_	nd	nd	nd	nd	nd	nd	nd	nd	nd	nd	nd	nd	18.9 ± 0.6 ^d^	nd	12.3 ± 0.5 ^a^
AppMP_LUHS29_	nd	nd	nd	nd	nd	nd	nd	nd	nd	20.1 ± 0.4 ^e^	nd	nd	11.2 ± 0.1 ^a^	nd	15.2 ± 0.3 ^b^
AppMP_LUHS122_	nd	nd	12.0 ± 0.4 ^a^	nd	nd	11.3 ± 0.2^a^	nd	nd	11.6 ± 0.6 ^b^	nd	nd	nd	nd	nd	20.9 ± 0.6 ^d^
AppMP_LUHS71_	nd	nd	nd	nd	nd	12.4 ± 0.5^b^	nd	nd	10.4 ± 0.4 ^a^	nd	nd	nd	nd	nd	17.6 ± 0.4 ^c^
AppMP_LUHS244_	nd	nd	12.3 ± 0.5 ^a b^	nd	nd	nd	nd	nd	11.2 ± 0.3 ^b^	nd	nd	nd	nd	nd	20.4 ± 0.3 ^d^
AppMP_LUHS183_	nd	nd	13.4 ± 0.6 ^b^	nd	nd	nd	nd	nd	12.9 ± 0.7 ^c^	nd	nd	nd	nd	nd	17.9 ± 0.8 ^c^
AppMP_LUHS206_	nd	nd	11.6 ± 0.2 ^a^	nd	nd	nd	nd	nd	11.2 ± 0.3 ^b^	nd	nd	nd	nd	nd	20.2 ± 0.4 ^d^
AppMPNF	nd	nd	nd	nd	nd	nd	nd	nd	nd	14.3 ± 0.6	nd	nd	10.8 ± 0.3	nd	16.6 ± 0.3 ^c^

Supercripts^a–e^—Mean values with different letters between a lines are significantly different (*p* ≤ 0.05). nd—not detected. MP—milk permeate; MP_LUHS245_—fermented with LUHS245 (*L. uvarum)*; MP_LUHS210_—fermented with LUHS210 (*L. casei)*; MP_LUHS51_—fermented with LUHS51 (*L. curvatus)*; MP_LUHS135_—fermented with LUHS135 (*L. plantarum)*; MP_LUHS29_—fermented with LUHS29 (*P. acidilactici)*; MP_LUHS122_—fermented with LUHS122 (*L. plantarum)*; MP_LUHS71_—fermented with LUHS71 (*L. coryniformins)*; MP_LUHS244_—fermented with LUHS244 (*L. paracasei)*; MP_LUHS183_—fermented with LUHS183 (*P. pentosaceus);* MP_LUHS206_—fermented with LUHS206 (*L. faraginis)*; MP_NF_—non-fermented.; App—with 8% (*w*/*w*) of apple by-products. 1—*Klebsiella pneumonia; 2*—*Salmonella enterica; 3*—*Pseudomonas aeruginosa; 4*—*Acinetobacter baumannii; 5*—*Proteus mirabilis; 6*—MRSA M87fox; 7—*Enterococcus faecalis; 8*—*Enterococcus faecium; 9*—*Bacillus cereus; 10*—*Streptococcus mutans; 11*—*Enterobacter cloacae; 12*—*Citrobacter freundii; 13*—*Streptococcus epidermis; 14*—*Staphylococcus haemolyticus; 15*—*Pasteurella multocida.*

**Table 3:** Average values and standard deviations (Mean ± STDV) (*n* = 3) of the antimicrobial activities in the milk permeate (MP) (0 h) and final fermented beverages (24 h), without and with addition of apple by-products, against 15 pathogenic and opportunistic microbial bacterial in liquid medium (+ indicates pathogen growth; - indicates that pathogen growth was not established).

	Growth ( + ) or Growth Absence (-) of Pathogenic and Opportunistic Bacteria															
	Pathogenic and Opportunistic Bacterial Strains															
Samples	*1*	*2*	*3*	*4*	*5*	6	*7*	*8*	*9*	*10*	*11*	*12*	*13*	*14*	*15*	Number of the Inhibited Pathogens
	*Without apple by-products* *Experimental design: 0.5 mL tested sample + 0.1 mL pathogen*															
MP_LUHS245_	-	-	-	-	-	-	+	+	-	-	-	-	-	-	-	13
MP_LUHS210_	-	-	-	-	-	-	+	+	-	-	-	-	-	-	-	13
MP_LUHS51_	-	-	-	-	+	-	+	+	-	-	+	-	-	-	-	11
MP_LUHS135_	+	+	+	+	+	-	+	+	-	-	+	+	-	-	-	6
MP_LUHS29_	+	-	-	-	-	-	+	+	-	-	-	-	-	-	-	12
MP_LUHS122_	+	-	-	-	-	-	+	+	-	-	-	-	-	-	-	12
MP_LUHS71_	+	-	-	-	-	-	+	+	-	-	-	-	-	-	-	12
MP_LUHS244_	+	-	-	-	-	-	+	+	-	-	+	-	-	-	-	11
MP_LUHS183_	+	-	-	-	-	-	+	+	-	-	-	-	-	-	-	12
MP_LUHS206_	+	-	-	-	-	-	+	+	-	-	-	-	-	-	-	12
MPNF	+	+	+	+	-	-	+	+	+	+	+	+	+	+	+	0
Pathogen control	+	+	+	+	+	+	+	+	+	+	+	+	+	+	+	-
LAB control	+	+	+	+	+	+	+	+	+	+	+	+	+	+	+	-
	*With apple by-products* *Experimental design: 0.5 mL tested sample + 0.1 mL pathogen*															
AppMP_LUHS245_	-	+	-	-	+	+	-	+	-	-	+	-	-	-	-	10
AppMP_LUHS210_	-	-	-	-	-	+	+	+	-	-	-	-	-	-	+	11
AppMP_LUHS51_	+	+	-	-	+	+	+	+	-	-	-	-	-	-	-	9
AppMP_LUHS135_	-	+	-	-	+	+	+	+	-	-	+	-	-	-	-	9
AppMP_LUHS29_	-	-	-	-	-	+	-	+	-	-	+	-	-	-	-	12
AppMP_LUHS122_	-	-	-	-	-	+	+	+	-	-	-	+	-	-	-	11
AppMP_LUHS71_	-	-	-	-	-	-	+	+	-	-	-	-	-	-	-	13
AppMP_LUHS244_	-	-	-	-	+	-	+	+	-	-	+	-	-	+	-	10
AppMP_LUHS183_	+	-	-	-	+	+	+	+	-	-	+	-	-	-	-	9
AppMP_LUHS206_	+	-	-	-	+	+	+	+	-	-	-	-	-	-	-	10
AppMPNF	+	-	-	-	+	+	-	+	-	-	+	-	-	+	-	9
Pathogen control	+	+	+	+	+	+	+	+	+	+	+	+	+	+	+	-
LAB control	+	+	+	+	+	+	+	+	+	+	+	+	+	+	+	-

MP—milk permeate; MP_LUHS245_—fermented with LUHS245 (*L. uvarum)*; MP_LUHS210_—fermented with LUHS210 (*L. casei)*; MP_LUHS51_—fermented with LUHS51 (*L. curvatus)*; MP_LUHS135_—fermented with LUHS135 (*L. plantarum)*; MP_LUHS29_—fermented with LUHS29 (*P. acidilactici)*; MP_LUHS122_—fermented with LUHS122 (*L. plantarum)*; MP_LUHS71_—fermented with LUHS71 (*L. coryniformins)*; MP_LUHS244_—fermented with LUHS244 (*L. paracasei)*; MP_LUHS183_—fermented with LUHS183 (*P. pentosaceus)*; MP_LUHS206_—fermented with LUHS206 (*L. faraginis)*; MPNF—non-fermented.; App—with 8% (*w*/*w*) of apple by-products. 1—*Klebsiella pneumonia; 2*—*Salmonella enterica; 3*—*Pseudomonas aeruginosa; 4*—*Acinetobacter baumannii; 5*—*Proteus mirabilis; 6*—MRSA M87fox; 7—*Enterococcus faecalis; 8*—*Enterococcus faecium; 9*—*Bacillus cereus; 10*—*Streptococcus mutans; 11*—*Enterobacter cloacae; 12*—*Citrobacter freundii; 13*—*Streptococcus epidermis; 14*—*Staphylococcus haemolyticus; 15*—*Pasteurella multocida.*

**Table 4:** Average values and standard deviations (Mean ± STDV) (*n* = 3) of the overall acceptability and emotions induced by the milk permeate (MP) (0 h) and final fermented beverages (24 h), without and with addition of apple by-products.

Beverages Samples	Overall Acceptability	Emotions Induced by the Beverages (from 0 to 1)								
		Neutral	Happy	Sad	Angry	Surprised	Scared	Disgusted	Contempt	Valence
	*Without apple by-products*									
MP_LUHS245_	3.9 ± 0.12 ^a^	0.42 ± 0.01 ^d^	0.06 ± 0.001 ^a^	0.24 ± 0.01 ^c^	0.08 ± 0.002 ^a^	0.03 ± 0.001 ^a^	0.0009 ± 0.00002 ^a^	0.0009 ± 0.00002 ^a^	0.01 ± 0.0002 ^a^	0.19 ± 0.004 ^c^
MP_LUHS210_	4.4 ± 0.13 ^b^	0.38 ± 0.01 ^c^	0.04 ± 0.001 ^a^	0.2 ± 0.004 ^b^	0.09 ± 0.002 ^a^	0.03 ± 0.001 ^a^	0.001 ± 0.00002 ^a^	0.001 ± 0.00002 ^a^	0.01 ± 0.0002 ^a^	0.19 ± 0.004 ^c^
MP_LUHS51_	4.3 ± 0.12 ^b^	0.28 ± 0.01 ^a^	0.09 ± 0.002 ^a^	0.18 ± 0.004 ^a^	0.06 ± 0.001 ^a^	0.04 ± 0.001 ^a^	0.001 ± 0.00002 ^a^	0.001 ± 0.00002 ^a^	0.02 ± 0.0004 ^a^	0.18 ± 0.004 ^c^
MP_LUHS135_	4.9 ± 0.12 ^c^	0.35 ± 0.01 ^c^	0.12 ± 0.002 ^b^	0.16 ± 0.003 ^a^	0.09 ± 0.002 ^a^	0.03 ± 0.001 ^a^	0.001 ± 0.00002 ^a^	0.001 ± 0.00002 ^a^	0.01 ± 0.0002 ^a^	0.22 ± 0.004 ^d^
MP_LUHS29_	5.3 ± 0.13 ^c^	0.23 ± 0.004 ^a^	0.14 ± 0.003 ^b^	0.16 ± 0.003 ^a^	0.13 ± 0.003 ^b^	0.01 ± 0.0002 ^a^	0.001 ± 0.00002 ^a^	0.001 ± 0.00002 ^a^	0.03 ± 0.0006 ^b^	0.13 ± 0.003 ^b^
MP_LUHS122_	4.5 ± 0.11 ^b^	0.39 ± 0.01 ^c^	0.03 ± 0.001 ^a^	0.15 ± 0.003 ^a^	0.1 ± 0.002 ^a^	0.04 ± 0.001 ^a^	0.01 ± 0.0002 ^b^	0.001 ± 0.00002 ^a^	0.02 ± 0.0004 ^a^	0.11 ± 0.002 ^a^
MP_LUHS71_	4.8 ± 0.08 ^c^	0.43 ± 0.01 ^d^	0.16 ± 0.0032 ^c^	0.08 ± 0.002 ^a^	0.03 ± 0.001 ^a^	0.02 ± 0.0004 ^a^	0.001 ± 0.00002 ^a^	0.001 ± 0.00002 ^a^	0.01 ± 0.0002 ^a^	0.13 ± 0.003 ^b^
MP_LUHS244_	3.9 ± 0,07 ^a^	0.34 ± 0.01 ^b^	0.18 ± 0.004 ^c^	0.11 ± 0.002 ^a^	0.07 ± 0.001 ^a^	0.01 ± 0.0002 ^a^	0.001 ± 0.00002 ^a^	0.001 ± 0.00002 ^a^	0.01 ± 0.0002 ^a^	0.09 ± 0.002 ^a^
MP_LUHS183_	5.1 ± 0.1 ^c^	0.31 ± 0.01 ^b^	0.16 ± 0.003 ^c^	0.2 ± 0.004 ^b^	0.05 ± 0.001 ^a^	0.01 ± 0.0002 ^a^	0.001 ± 0.00002 ^a^	0.002 ± 0.00004 ^a^	0.02 ± 0.0004 ^a^	0.29 ± 0.01 ^e^
MP_LUHS206_	3.8 ± 0,08 ^a^	0.34 ± 0.01 ^b^	0.17 ± 0.0034 ^c^	0.17 ± 0.003 ^a^	0.12 ± 0.002 ^b^	0.06 ± 0.0012 ^b^	0.002 ± 0.00004 ^a^	0.001 ± 0.00002 ^a^	0.03 ± 0.001 ^b^	0.18 ± 0.004 ^c^
MPNF	3.5 ± 0.07 ^a^	0.21 ± 0.004 ^a^	0.19 ± 0.004 ^c^	0.19 ± 0.004 ^a^	0.09 ± 0.002 ^a^	0.01 ± 0.0002 ^a^	0.001 ± 0.00002 ^a^	0.001 ± 0.00002 ^a^	0.09 ± 0.002 ^c^	0.22 ± 0.004 ^d^
	*With apple by-products*									
AppMP_LUHS245_	5.3 ± 0.08 ^c^	0.45 ± 0.01 ^d^	0.08 ± 0.002 ^a^	0.16 ± 0.003 ^a^	0.1 ± 0.002 ^a^	0.02 ± 0.0004 ^a^	0.001 ± 0.00002 ^a^	0.001 ± 0.00002 ^a^	0.11 ± 0.002 ^c^	0.18 ± 0.004 ^c^
AppMP_LUHS210_	5.8 ± 0.09 ^d^	0.32 ± 0.01 ^b^	0.01 ± 0.0002 ^a^	0.43 ± 0.009	0.05 ± 0.001 ^a^	0.03 ± 0.001 ^a^	0.001 ± 0.00002 ^a^	0.001 ± 0.00002 ^a^	0.13 ± 0.003 ^d^	0.27 ± 0.01 ^e^
AppMP_LUHS51_	5.9 ± 0.08 ^d^	0.29 ± 0.01 ^a^	0.02 ± 0.0004 ^a^	0.22 ± 0.004 ^b^	0.16 ± 0.003 ^c^	0.04 ± 0.001 ^a^	0.001 ± 0.00002 ^a^	0.001 ± 0.00002 ^a^	0.09 ± 0.002 ^c^	0.25 ± 0.005 ^e^
AppMP_LUHS135_	5.5 ± 0.08 ^c^	0.3 ± 0.01 ^b^	0.02 ± 0.0004 ^a^	0.29 ± 0.006 ^c^	0.14 ± 0.003 ^b^	0.02 ± 0.0004 ^a^	0.001 ± 0.00002 ^a^	0.001 ± 0.00002 ^a^	0.08 ± 0.0016 ^c^	0.25 ± 0.005 ^e^
AppMP_LUHS29_	5.9 ± 0.06 ^d^	0.24 ± 0.01 ^a^	0.07 ± 0.0014 ^a^	0.28 ± 0.006 ^c^	0.21 ± 0.004 ^d^	0.04 ± 0.001 ^a^	0.001 ± 0.00002 ^a^	0.001 ± 0.00002 ^a^	0.12 ± 0.002 ^d^	0.38 ± 0.01^f^
AppMP_LUHS122_	6.1 ± 0.09 ^d^	0.26 ± 0.01 ^a^	0.05 ± 0.001 ^a^	0.24 ± 0.01 ^b^	0.17 ± 0.003 ^c^	0.01 ± 0.0002 ^a^	0.001 ± 0.00002 ^a^	0.001 ± 0.00002 ^a^	0.11 ± 0.002 ^c^	0.18 ± 0.004 ^c^
AppMP_LUHS71_	6.4 ± 0.09 ^d^	0.36 ± 0.01 ^b^	0.06 ± 0.001 ^a^	0.16 ± 0.003 ^a^	0.09 ± 0.002 ^a^	0.02 ± 0.0004 ^a^	0.001 ± 0.00002 ^a^	0.001 ± 0.00002 ^a^	0.09 ± 0.002 ^c^	0.16 ± 0.003 ^b^
AppMP_LUHS244_	6.8 ± 0.23 ^e^	0.33 ± 0.01 ^b^	0.07 ± 0.001 ^a^	0.15 ± 0.003 ^a^	0.1 ± 0.002 ^a^	0.01 ± 0.0002 ^a^	0.001 ± 0.00002 ^a^	0.001 ± 0.00002 ^a^	0.05 ± 0.001 ^b^	0.15 ± 0.003 ^b^
AppMP_LUHS183_	7.1 ± 0.25 ^e^	0.38 ± 0.02 ^c^	0.04 ± 0.001 ^a^	0.13 ± 0.003 ^a^	0.1 ± 0.002 ^a^	0.08 ± 0.002 ^b^	0.005 ± 0.0001 ^a^	0.002 ± 0.00004 ^a^	0.03 ± 0.001 ^b^	0.16 ± 0.003 ^b^
AppMP_LUHS206_	6.5 ± 0.23 ^d e^	0.36 ± 0.02 ^c^	0.08 ± 0.002 ^a^	0.16 ± 0.003 ^a^	0.09 ± 0.002 ^a^	0.01 ± 0.0002 ^a^	0.001 ± 0.00002 ^a^	0.001 ± 0.00002 ^a^	0.01 ± 0.0002 ^a^	0.14 ± 0.003 ^b^
AppMP_NF_	5.2 ± 0.18 ^c^	0.37 ± 0.02 ^c^	0.13 ± 0.003 ^b^	0.18 ± 0.004 ^a^	0.06 ± 0.001 ^a^	0.03 ± 0.001 ^a^	0.001 ± 0.00002 ^a^	0.001 ± 0.00002 ^a^	0.09 ± 0.002 ^c^	0.08 ± 0.002 ^a^

Supercripts^a–f^—Mean values with different letters between a lines are significantly different (*p* ≤ 0.05). nd—not detected. MP—milk permeate; MP_LUHS245_—fermented with LUHS245 (*L. uvarum)*; MP_LUHS210_—fermented with LUHS210 (*L. casei)*; MP_LUHS51_—fermented with LUHS51 (*L. curvatus)*; MP_LUHS135_—fermented with LUHS135 (*L. plantarum)*; MP_LUHS29_—fermented with LUHS29 (*P. acidilactici)*; MP_LUHS122_—fermented with LUHS122 (*L. plantarum)*; MP_LUHS71_—fermented with LUHS71 (*L. coryniformins)*; MP_LUHS244_—fermented with LUHS244 (*L. paracasei)*; MP_LUHS183_—fermented with LUHS183 (*P. pentosaceus)*; MP_LUHS206_—fermented with LUHS206 (*L. faraginis)*; MPNF—non-fermented.; App—with 8% (*w*/*w*) of apple by-products.

## Abbreviations

ACN	Acetonitrile
App	Apple by-products
CO_2_	Carbon dioxide
log_10_(CFU/mL)	Colony-forming units per mL of sample
DIZ	Diameter of inhibition zones
EDTA-2Na	Ethylenediamine tetraacetic acid disodium salt
ELSD	Evaporative Light-Scattering Detector
GOS	Galactooligosaccharides
HPAEC-PAD	High-Performance Anion-Exchange Chromatography with Pulsed Amperometric Detection
HPLC	High-performance liquid chromatography
HPLC-ELSD	High-performance liquid chromatography with Evaporative Light-Scattering Detection
I.D.	Internal diameter
LAB	Lactic acid bacteria
MP	Milk permeate
AppMP	with Apple by-products
MH	Mueller–Hinton
°N	Neiman degrees
HNO_3_	Nitric acid
NP	Normal-phase
nd	Not detected
1-way ANOVA	One-way analysis of variance
OA	Overall acceptability
E	Potential
STDV	Standard deviation
The Man Rogosa and Sharpe	MRS
Time periods	*t*
Total titratable acidity	TTA
Water	H_2_O
